# Job Burnout on Subjective Well-Being Among Chinese Female Doctors: The Moderating Role of Perceived Social Support

**DOI:** 10.3389/fpsyg.2020.00435

**Published:** 2020-03-31

**Authors:** Liping Wang, Huiping Wang, Shuhong Shao, Gaizhen Jia, Jing Xiang

**Affiliations:** ^1^Department of Education Science, Ludong University, Yantai, China; ^2^Department of Applied Psychology, Binzhou Medical University, Yantai, China

**Keywords:** female doctor, job burnout, subjective wellbeing, perceived social support, family support

## Abstract

All doctors face challenges and pressures that can lead to job burnout. The dual pressures of work and family make female doctors less happy and more likely to experience burnout, but few studies have focused on female doctors. In this study we explore the influence of job burnout on female clinical doctors’ subjective wellbeing and the moderating role of perceived social support. A casual comparative study design was used for this research. Three self-reporting scales (Maslach Burnout Inventory, Perceived Social Support Scale and Subjective Wellbeing Scale) were distributed to participants, who were selected through random sampling. Participants consisted of 120 female and 120 male doctors from a hospital of Yantai City. Female doctors scored significantly higher than male doctors in the emotional exhaustion dimension (*p* < 0.01), and female doctors’ subjective wellbeing was lower than that of male doctors (*p* < 0.01). The three dimensions of job burnout and subjective wellbeing exhibited significant negative correlations, and a positive relationship was found between perceived social support and subjective wellbeing in female doctors (*p* < 0.01). Perceived social support, especially from family, played a moderating role between emotional exhaustion and subjective wellbeing, and the moderating effect was significant (*p* < 0.01). A significant difference was observed between male and female doctors; female doctors experienced more emotional exhaustion and lower subjective wellbeing than male doctors. Improving perceived social support could reduce burnout and enhance subjective wellbeing.

## Introduction

As the proportion of women in the workforce continues to rise, women’s health has become an issue of global concern. Due to differing social roles of men and women, women face dual pressures of work and family, and their mental health can become compromised. A survey from China Social Sciences Academic Press found that 87% of women in China experienced work stress, and more than half reported heavy stress. After the demands of daily work, the difficulty of balancing work and family has become the greatest source of stress (Chinese Women’s Living Conditions Report, 2017). Another survey demonstrated that among 10,000 married women employed in Beijing, those from 25 to 45 years old (80.75%) experienced high levels of pressure (Li et al., 2006). Female clinical doctors work in the front line of the medical industry, directly facing the patients. Gender and occupation characteristics suggest that they suffer more stress and have a higher risk of job burnout than men in similar positions.

Job burnout is a psychological syndrome, a long-term response to chronic interpersonal stress at work. It was first measured by Maslach in 1981 and defined as a state of emotional exhaustion (EE), depersonalization (DP) and low personal accomplishment (LPA). Emotional exhaustion is described by reduced energy levels and extreme fatigue, as well as wearing out, loss of energy, depletion and debilitation (Maslach et al., 2016). Depersonalization, also called cynicism, refers to a negative or inappropriate attitude toward clients, including irritability, loss of idealism, and withdrawal. Low personal accomplishment is indicated by a decrease in personal achievement, also called inefficacy, and described as reduced productivity or capability, low morale and an inability to cope (Maslach and Leiter, 2016).

Previous studies have revealed that female clinical doctors are more vulnerable to burnout than their male colleagues and found the presented symptoms to be more prominent in women. Further, the prevalence of burnout syndrome has been reported as higher in female academic physicians (Moore et al., 2019). A survey measuring doctors’ working state done by the Association of American Physicians showed that the rate of job burnout in female doctors is two times that of male doctors (Rabatin et al., 2016). Human Resources Development in China released a survey reporting the job burnout rate of women at 41.38%, significantly higher than that of men (37.23%). Amanda C. North (2017) found that female physicians were more susceptible to burnout than their male colleagues. Atefeh Soltanifar et al. (2018) reported that in Iran 94.8% of female emergency medicine physicians perceived their workload to be moderate to high, and only 1.3% of them experienced high job satisfaction (Soltanifar et al., 2018). Győrffy et al. (2016) revealed that women’s workload increased in 10 years from 2003 to 2013, but job satisfaction decreased, women perceived more stressful or difficult work-related situations, and sense of personal accomplishment significantly decreased.

Payne and Fletcher (1983) put forward the demand-supports-constraints model, which suggests that individual constraints on support and resources lead to stress. Social support can reduce stress at work. If one perceives support from family, friends and colleagues, they tend to feel more life satisfaction and less burnout. Previous research has revealed that job-related relationships characterized by a lack of support and trust increased the risk of burnout (Frogeli et al., 2019). In contrast, when these job-related relationships are positive and there is a high level of social support, employees have effective means of working out disagreements and are more likely to experience job engagement. At the same time, it has been found that support from superiors reduces burnout more than support from colleagues (Maslach et al., 2001).

Supervisory support may help prevent turnover through reduction of work-related stress (Fukui et al., 2019). Job requirements resource theory suggests that job resources are negatively correlated with job burnout. When individuals are supported by their work groups, job resources become enriched, and job resources can effectively reduce the prevalence of job burnout (Bernabe and Botia, 2016; Fukui et al., 2019). Job burnout has become an important factor affecting individual subjective wellbeing. As the individual’s standard for overall quality of life evaluation, subjective wellbeing is a comprehensive indicator of the main reaction to social function and adaptation, subjectivity, integrity, relative stability and other characteristics. Studies have shown that perceived social support (subjective support) is likely to be beneficial to an individual’s mental health, including wellbeing (Lian and Guo, 2017). Studies on teachers have found that social support plays a partial mediating role in the relationship between teachers’ job burnout and life satisfaction (Yang et al., 2015).

Recently, studies on the subjective wellbeing of people around the world have mainly focused on young students and particular groups such as civil servants, the elderly, teachers, and nurses. There are few studies on doctors, especially female doctors. The current study explores job burnout, perceived social support and subjective wellbeing in female doctors. Our hypothesis is that job burnout can reduce subjective wellbeing, but perceived social support can reduce job burnout, and as a result can improve subjective wellbeing.

Based on the literature, the research hypotheses are as follows:

H1. There are differences in job burnout and subjective wellbeing between male and female doctors.

H2. There is a negative relationship between job burnout and subjective wellbeing in female doctors.

H3. There is a positive relationship between perceived social support and subjective wellbeing in female doctors.

H4. There is a negative relationship between job burnout and perceived social support in female doctors.

H5. Perceived social support moderates the relationship between job burnout and subjective wellbeing in female doctors.

## Materials and Methods

This cross-sectional survey was carried out on Chinese female clinicians. Using a casual comparative design, the data were analyzed based on different departments in the hospital, and the random sampling method was applied to select participants; students in psychology were given paper tests during doctors’ succession meetings. The investigation was conducted anonymously and with the oral consent of the clinician; they were assured that participation was voluntary and that all data would remain confidential and unidentified.

### Participants

From November 2017 to April 2018, 120 female doctors from a hospital of Yantai participated. The total number of medical staff is 1400, including 580 doctors and 820 paramedics. In this study, average age of participating female doctors was 31.73 ± 4.95 years and average work time 8.36 ± 5.75 h. The study also selected 120 male doctors for comparison; their average age was 32.97 ± 5.01 years and average work time 9.41 ± 8.76 h. Participants completed three self-report questionnaires: 226 effective questionnaires in total, and the effective rate was 94.17% (Demographic data in Table 1).

**TABLE 1:** Demographic data table.

Variable	Female doctor (*n*/%)	Male doctor (*n*/%)	*x^2^/t*	*p*
Average age	31.73 ± 4.95	32.97 ± 5.01	*t* = 1.874	0.061
**Department**				
Internal medicine	40 (35.7%)	30 (26.3%)	*x^2^* = 7.346	0.062
Surgery	38 (33.9%)	53 (46.5%)		
Care department	10 (8.9%)	16 (14.0%)		
The other	24 (21.4%)	15 (13.1%)		
**Education**				
Junior college	11 (9.8%)	6 (5.3%)	*x^2^* = 4.465	0.107
Bachelor	71 (63.4%)	64 (56.1%)		
Master degree	30 (26.8%)	44 (38.6%)		
**Marital status**				
Unmarried	30 (26.8%)	22 (19.3%)	*x^2^* = 0.291	0.233
Married	82 (73.2%)	92 (80.7%)		
Average work years	8.36 ± 5.75	9.41 ± 8.76	*t* = 1.07	0.286
**Work experience**				
0–4	40 (35.7%)	32 (28.1%)		
5–9	18 (16.1%)	44 (38.6%)		
10–19	50 (44.6%)	24 (21.1%)		
>19	4 (3.6%)	14 (12.3%)		

### Questionnaires

#### Maslach Burnout Inventory

The questionnaire was based on the Maslach Burnout Inventory-General Survey (MBI-GS), revised to be suitable for Chinese people by Li and Shi (2003), including three parts: emotional exhaustion (EE), depersonalization (DP) and low personal accomplishment (LPA). The EE subscale consists of five items; the DP subscale consists of four, and the LPA scale consists of six questions; LPA requires reverse scoring. Using the Likert 7-point system, a scale from 0 to 6 in which 0 represents “never” and 6 “every day,” the internal consistency coefficients in this study were 0.932, 0.750, and 0.839, which meet the survey requirements. The score of each subscale was calculated by summing the scores of items.

#### Perceived Social Support Scale

The perceived social support scale (PSSS) was developed by Zimet et al. (1988). We used the Chinese version, revised by domestic scholar Jang (1999), to measure the degree of perceived social support. There are 12 items in the scale, including family support (FAS), friend support (FRS) and other support (OS). The items are measured using a 5-point scale from 1 (less support) to 5 (more support). Thus, the higher the score, the greater the support. In this study, the internal consistency reliability coefficient of the scale was 0.93.

#### Subjective Wellbeing Scale

The subjective wellbeing scale (SWB) is the testing tool for health statistics of the United States National Center, which aims to evaluate subjects’ statements about happiness. Revised by Duan (1996), the scale consists of 18 items. The internal consistency coefficient in this study was 0.771.

### Data Analysis

The SPSS 21 statistical program was used to perform descriptive analyses, difference analysis, and correlation analysis. Structural equations were calculated using the application Mplus 8.0, with job burnout as an independent variable, perceived social support as a moderating variable and subjective wellbeing as a dependent variable, to explore the moderating effect of perceived social support (Baron and Kenny, 1986; Wen et al., 2012; Hayes, 2013; Wang, 2017).

## Results

### The Basic Data

The result of the chi-square test revealed no significant differences between departments (*x*^2^ = 7.346, *p* = 0.062), no significant differences in terms of the level of education (*x*^2^ = 4.465, *p* = 0.107) and no significant differences in terms of marital status (*x*^2^ = 0.291, *p* = 0.233). There were no significant differences in average age (*t* = 1.874, *p* = 0.061) and average work time (*t* = 1.07, *p* = 0.286) between female and male doctors.

### Gender Differences

Table 2 shows that the level of EE in female doctors was significantly higher than that of male doctors (*p* < 0.01); effect size was 0.620. According to Cohen (1988), 0.5 is a middle effect size. The effect size of gender in the dimension of EE reaches the middle level. Subjective wellbeing was significantly lower in female doctors than in male doctors (*p* < 0.01), and effect size was 0.438, which is a low effect level. However, in terms of the sense of achievement, female doctors scored significantly higher than male doctors (*p* < 0.01); effect size was 0.400, which is also a low effect level. The results show significant differences in job burnout and subjective wellbeing between male and female doctors, except for the DP dimension, partly supporting the H1 hypothesis.

**TABLE 2:** The *t*-test of different gender doctors’ job burnout and SWB.

Variable	Female	Male	*t*	*p*	*ES*
EE	19.54 ± 5.77	15.82 ± 6.19	4.661	0.000**	0.620
DP	12.18 ± 4.40	12.18 ± 3.69	0.006	0.995	0.0008
LPA	14.14 ± 4.85	16.28 ± 4.91	–3.293	0.001**	0.438
SWB	68.27 ± 11.21	73.25 ± 13.54	–3.008	0.003**	0.400

EE: emotional exhaustion; DP: depersonalization; LPA: low personal accomplishment; SWB: subjective wellbeing; ES: effect size **p<0.01.

### The Correlation Analysis

This study focuses on job burnout and subjective wellbeing of female doctors. After the gender comparison, relevant indicators of female doctors are further discussed. Finally, the statistical analysis of data from female doctors is presented. Table 3 shows that the three dimensions of job burnout and subjective wellbeing were negatively correlated (*r* = −0.435, −0.356, −0.233, −0.460, *p* < 0.01), which confirms hypothesis H2. Statistically significant correlations were observed between perceived social support and subjective wellbeing except in the dimension of FRS (*r* = 0.248, 0.218, 0.328, *p* < 0.01), which confirms hypothesis H3. Job burnout and perceived social support were negatively correlated (*r* = −0.356, −0.283, −0.275, −0.446, *p* < 0.01), but there were no statistically significant correlations between DP and perceived social support, FAS or FRS (*p* > 0.05), partially confirming H4.

**TABLE 3:** Correlation analysis of job burnout of female doctors and SWB.

	EE	DP	LPA	FAS	FRS	OS	PSS	SWB
EE	1							
DP	0.447**	1						
LPA	0.174	0.460**	1					
FAS	–0.176	–0.078	−0.380**	1				
FRS	–0.167	–0.105	−0.348**	0.878**	1			
OS	−0.321**	−0.263**	−0.421**	0.714**	0.687**	1		
PSS	−0.234*	–0.153	−0.414**	0.950**	0.942**	0.856**	1	
SWB	−0.435**	−0.356**	−0.233**	0.218**	0.157	0.328**	0.248**	1

EE: emotional exhaustion; DP: depersonalization; LPA: low personal accomplishment; JBO: the total score of job burnout; SWB: subjective wellbeing; PSS: perceived social support; FAS: family support; FRS: friend support; OS: other support. *p < 0.05, **p < 0.01.

### Moderating Effect

**FIGURE 1:**
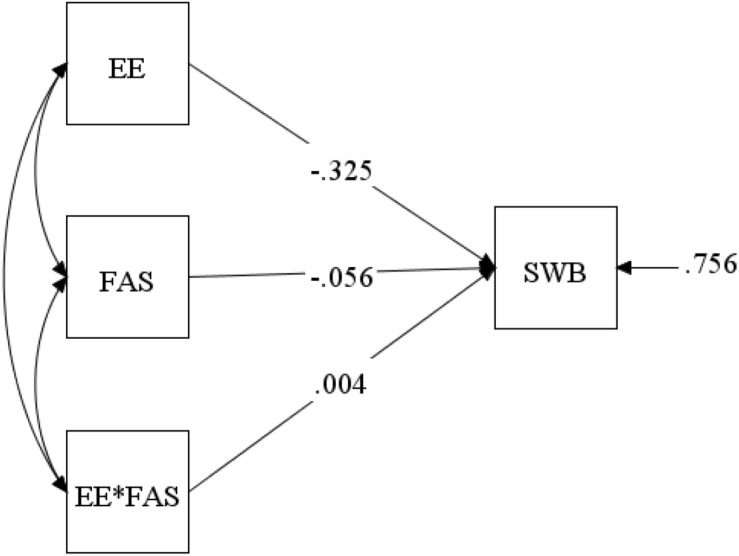
A moderate model of emotional exhaustion (EE) and subjective wellbeing (SWB) through family support (one dimension of perceived social support). Standardized regression coefficients are provided along the paths. EE: emotional exhaustion; SWB: subjective wellbeing; FAS: family support.

**FIGURE 2:**
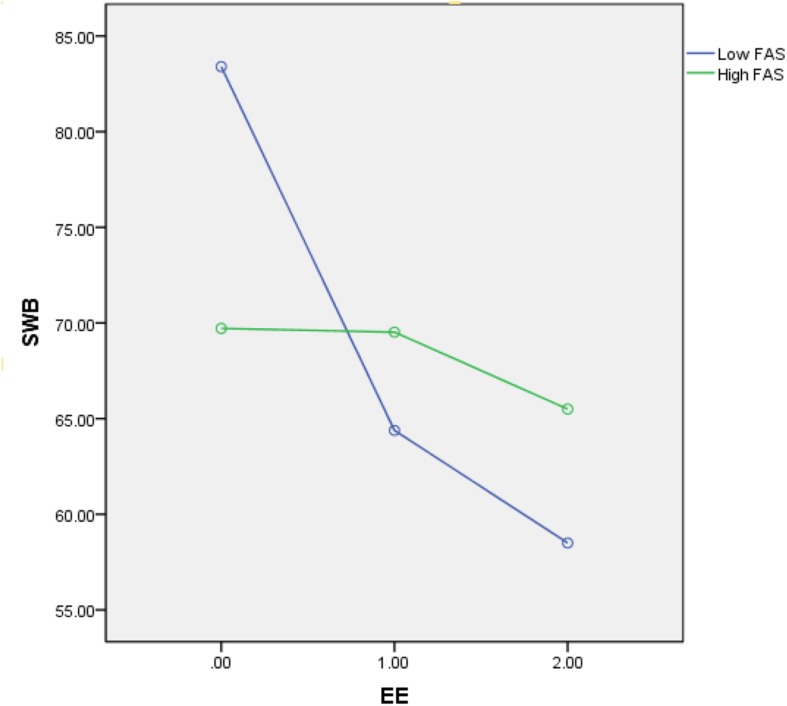
The interaction between emotional exhaustion and family support on subject wellbeing (0.00 represented low group; 1.00 represented moderate group; 2.00 represented severe group). EE: emotional exhaustion; SWB: subjective wellbeing; FAS: family support.

To investigate H5 – that perceived social support moderates the relationship between job burnout and subjective wellbeing in female doctors – the moderate effect path diagram was established. According to the method of Wen et al. (2012), we verified every model in Mplus. The three dimensions of job burnout (EE, DP, and LPA) were used as independent variables, the three dimensions of perceived social support (FAS, FRS, and OS) as moderating variables and subjective wellbeing as dependent variables, in order to assess the moderating effect of perceived social support. Decentralization treatment of the data in Mplus revealed that the interaction effect of FAS and EE was significant (Path analysis is shown in Figure 1), but the same was not true for FRS and OS. The main effect estimates for FAS were −0.056, *p* = 0.008 < 0.01; the interaction effect was 0.004, *p* = 0.000 < 0.01, as shown in Table 4. This suggests that the moderating effect of FAS was significant. Figure 2 illustrates the interaction between EE and FAS. According to the revised edition of MBI-GS (Li and Shi, 2003), scores less than 15 for EE were classified into a low group (represented by.00), 15–25 were classified into a moderate group (represented by 1.00), and those greater than 25 were classified into a severe group (represented by 2.00). This indicates that when female doctors feel EE, if they could get FAS, EE would be alleviated and subjective wellbeing would improve. FAS played an important buffer role.

**TABLE 4:** Moderating effect for the model.

SWB on	Estimate	S.E	*p*
EE	–0.325	0.071	0.000
FAS	–0.056	0.021	0.008
EE*FAS	0.004	0.001	0.000

## Discussion

In this study, a significant difference was observed between male and female doctors, supporting the H1 hypothesis. Female doctors reported more EE and lower subjective wellbeing than men, which confirms hypothesis H1. Recent studies have found that female doctors face more burnout. According to Soltanifar et al.’s (2018) study, 84% of female emergency medicine physicians suffered from moderate to high levels of EE, 48.1% had moderate to high levels of DP and 80.5% had moderate to high levels of burnout in the LPA subscale. Moore et al.’s (2019) survey indicates that a high percentage of female neurologists experience symptoms of burnout. In a study among primary care physicians, women were almost twice as likely as men to report burnout (Rabatin et al., 2016).

Clinicians experience high levels of pressure (Soltanifar et al., 2018). They work to save patients, cure them and to reduce pain. They consistently work long hours (Soltanifar et al., 2018; Mumbwe et al., 2019). Their work is not only related to life and death but also to a patients’ family happiness and even to a healthy and stable society. Previous studies have shown that women physicians tend to display more empathy and report especially high empathic concern for patients (Gleichgerrcht and Decety, 2013; Cho and Jeon, 2019). Moreover, women are more likely than male physicians to counsel their patients and spend more time with each patient (Barnett et al., 2019). They also shoulder the responsibility of taking care of their own families, raising children, caring for the elderly and so on. Many researchers have revealed that the work-family conflict can influence women’s burnout and subjective wellbeing (Kim and Windsor, 2015; Pu et al., 2017). In a survey of satisfaction with work–life balance, only 8.5% of women physicians indicated being very satisfied (Soltanifar et al., 2018). The modern female doctor undertakes the dual pressures of work and family so is more prone to job burnout and a compromised level of subjective wellbeing.

In our research, the three dimensions of job burnout and subjective wellbeing were negatively correlated, confirming H2. Statistically significant correlations were observed between perceived social support and subjective wellbeing except in the FRS dimension, confirming the H3. The two dimensions of job burnout (except DP) and perceived social support were negatively correlated, partially confirming H4. Barnett et al. (2019) found workplace social support, not personal social support, was associated with lower psychological distress; Sadaaki Fukui found (Fukui et al., 2019) that supervisory support had positive effects in reducing turnover intention through reduced EE. The study indicated that in the workplace the support from supervisors and colleagues was more important than that of family and friends (Fukui et al., 2019). We found no significant correlation between DP and perceived social support, but LPA and perceived social support were significantly correlated, and FAS, FRS, and OS were all related to female doctors’ LPA. The results suggest that if female doctors want to experience high personal accomplishment, they need more social support.

Past research has amply demonstrated the importance of social support, showing that it can affect a person’s health and wellbeing (Mumbwe et al., 2019; Popa-Velea et al., 2019). Our findings indicate that job burnout influences subjective wellbeing, and perceived social support plays a moderating role between job burnout and subjective wellbeing. We found that FAS played a moderating role between EE and subjective wellbeing, partly confirming H5. The moderating effect was small, but the results were significant (Wen et al., 2012; Wang, 2017). More precise hypotheses should be tested in future research. Female doctors’ emotion is more influenced by the doctor-patient relationship. Previous studies have shown that female doctors tend to show more empathy (Gleichgerrcht and Decety, 2013; Cho and Jeon, 2019) and offer patients more comfort and emotional care. In addition to the increased demands of work, family responsibilities and dissatisfaction with work-life balance also play an important role in the mood of female doctors (Beckett et al., 2015). Women feel more pressure at home (Ma, 2017), leading to higher levels of EE, leading to increased depression and anxiety (Khamisa et al., 2015; Lin et al., 2016). Previous studies have found that social support reduces emotional burnout (Blanco Donoso et al., 2017; Lian and Guo, 2017). This is consistent with our findings. We further found that FAS played a moderating role between EE and subjective wellbeing, but we did not find an interaction between social support in DP and LPA and subjective wellbeing. It may be that FAS has a greater impact on EE. The family is the source of human emotion, support from family is important to everybody; it can influence one’s health, both physically and psychologically (Uchmanowicz et al., 2019). Family support could positively predict subjective wellbeing (Moore et al., 2019). Given one more social support, FAS could reduce burnout of female doctors and enhance their subjective wellbeing.

Job burnout negatively impacts an individual’s physical and mental health, as well as their work, family and society. This is especially true for doctors, who not only experience decreased individual job performance and increased susceptibility to physical and mental diseases, but can also experience a decline in their patients’ degree of trust, reducing patient compliance and creating negative consequences. Therefore, it is important to give them more support. Prevention of job burnout for doctors can maintain good working conditions, improve the doctor’s subjective wellbeing and reduce medical disputes; at the same time, it can increase work efficiency, reduce medical loss, improve patient compliance and patient satisfaction and thereby increase social benefits.

### Limitations

This study was limited in several ways. The sample size was small, and participants were recruited from a single hospital. Additionally, the self-reporting of the measures could introduce issues related to bias and social desirability. Future studies could utilize longitudinal research designs to track the effects of social support and burnout and test more precise hypotheses, providing a clearer picture of how these variables interact.

## Conclusion

Female doctors’ job burnout was significantly higher than men’s in the EE dimension, and their subjective wellbeing was lower than men’s. The size of the difference between men and women was moderate in EE. The three dimensions of job burnout and subjective wellbeing were negatively correlated, and the size of these correlations was low to moderate. Family support played a small moderating role between EE and subjective wellbeing. Increased FAS could reduce EE and improve subjective wellbeing.

## Data Availability Statement

The datasets generated for this study are available on request to the corresponding author.

## Ethics Statement

The study was approved by the Ethics Committee of Binzhou Medical University. The investigation was anonymous and with the doctor’s verbal consent; they were assured that participation was voluntary and that all data would remain confidential and unidentified.

## Author Contributions

LW designed the work and drafted the work. HW contributed point of result analyses. SS revised the manuscript’s main results. GJ and JX mainly statistical analysis and data processing. All authors read and approved the final version of the manuscript and they agreed to be accountable for all aspects of the work.

## Conflict of Interest

The authors declare that the research was conducted in the absence of any commercial or financial relationships that could be construed as a potential conflict of interest.
